# New Mode of Operating for Nævus

**Published:** 1831-04-01

**Authors:** 


### VII. NEW MODE OF OPERATING FOR NÆVUS. By DR. M. HALL.

This operation consists in the introduction of a couching-needle with cutting edges, at one point of the circumference of the nævus, close by the adjoining healthy skin. From this point the instrument is made to pass through the tumour in eight or ten different directions, so as to produce slight incisions through its textures, parallel with the skin, but not so as to pierce the tumour in any other part. The first point of puncture is to be made the *centre* of several rays of slight incisions effected by merely withdrawing, and again pushing forward the instrument, in the manner and in the various directions already mentioned. This plan was practised, under Dr. Hall's observation, by Mr. Heming, of Kentish-town, on a nævus somewhat larger than a shilling. A little pressure was applied over the tumour—there was no pain—no hæmorrhage. For several weeks, nothing appeared to have been effected by the operation, and it was concluded that the operation had failed. "What a short time did not effect, however, a longer period accomplished completely. Half a year after the operation, the tumour was found to have disappeared, and the colour of the skin to be nearly natural."

As the operation was performed in only one instance, and, as a long time intervened between it and any sensible effect, we have some misgivings as to the "*post hoc, ergo propter hoc,*" on this occasion. We once got into some discredit by strenously recommending an operation for a nævus situated on the head of a child; but which operation was resisted by the tender-hearted mother. About a year afterwards the mother brought the child to us, when, to our surprize, the nævus had entirely disappeared, *without any operation.* We do not mention this fact to throw any discredit on Dr. Hall's proposal; but only to shew that *one instance* is but slender ground for drawing a conclusion, either in medicine or surgery.

